# Comparison of Proton and Photon Beam Irradiation in Radiation-Induced Intestinal Injury Using a Mouse Model

**DOI:** 10.3390/ijms20081894

**Published:** 2019-04-17

**Authors:** Changhoon Choi, Chansu Lee, Sung-Won Shin, Shin-Yeong Kim, Sung Noh Hong, Hee Chul Park

**Affiliations:** 1 Department of Radiation Oncology, Samsung Medical Center, Seoul 06351, Korea; chchoi93@gmail.com (C.C.); camuserik@gmail.com (S.-W.S.); syeong.kim@sbri.co.kr (S.-Y.K.); 2 Department of Medicine, Samsung Medical Center, Seoul 06351, Korea; cslhero@gmail.com; 3 Sungkyunkwan University School of Medicine, Seoul 06351, Korea

**Keywords:** proton beam therapy, relative biological effectiveness, intestinal stem cells, crypt regeneration

## Abstract

When radiotherapy is applied to the abdomen or pelvis, normal tissue toxicity in the gastrointestinal (GI) tract is considered a major dose-limiting factor. Proton beam therapy has a specific advantage in terms of reduced doses to normal tissues. This study investigated the fundamental differences between proton- and X-ray-induced intestinal injuries in mouse models. C57BL/6J mice were irradiated with 6-MV X-rays or 230-MeV protons and were sacrificed after 84 h. The number of surviving crypts per circumference of the jejunum was identified using Hematoxylin and Eosin staining. Diverse intestinal stem cell (ISC) populations and apoptotic cells were analyzed using immunohistochemistry (IHC) and a terminal deoxynucleotidyl transferase-mediated dUTP nick-end labelling (TUNEL) assay, respectively. The crypt microcolony assay revealed a radiation-dose-dependent decrease in the number of regenerative crypts in the mouse jejunum; proton irradiation was more effective than X-ray irradiation with a relative biological effectiveness of 1.14. The jejunum is the most sensitive to radiations, followed by the ileum and the colon. Both types of radiation therapy decreased the number of radiosensitive, active cycling ISC populations. However, a higher number of radioresistant, reserve ISC populations and Paneth cells were eradicated by proton irradiation than X-ray irradiation, as shown in the IHC analyses. The TUNEL assay revealed that proton irradiation was more effective in enhancing apoptotic cell death than X-ray irradiation. This study conducted a detailed analysis on the effects of proton irradiation versus X-ray irradiation on intestinal crypt regeneration in mouse models. Our findings revealed that proton irradiation has a direct effect on ISC populations, which may result in an increase in the risk of GI toxicity during proton beam therapy.

## 1. Introduction

Proton beam therapy (PBT) is a type of particle-based radiation therapy (RT) that is increasingly used; currently, there are more than 60 operating proton therapy facilities worldwide [1]. Proton therapy is preferred for the treatment of solid tumors due to one of its physical properties, namely the Bragg peak [2,3]. The modulation of the Bragg peak of protons allows for a conformal delivery of the radiation dose to the target tumors while saving the surrounding normal tissues. Accumulating evidence indicates that PBT is more beneficial than conventional RT and is advantageous particularly in patients with tumors that are located adjacent to critical organs.

During the initial setup for PBT machines in most facilities, the relative biological effectiveness (RBE) of protons must be determined as part of the biological quality assurance (QA) process [4,5]. RBE is defined as the ratio of the reference radiation (e.g., megavoltage X-rays) dose to the proton dose that yields the same biological output. Proton RBE varies depending on the radiation dose per fraction, linear energy transfer (LET), tissue types, biological end points, and so on [5,6,7]. Despite biological uncertainty, almost all PBT facilities use a fixed RBE value of 1.1, thereby ignoring the variability of proton RBE [6,8]. Various biological experiments, including an in vitro colony survival assay and in vivo intestine crypt survival assay, estimated a proton RBE of 1.1 [5].

The intestinal epithelium is an excellent model system for tissue homeostasis and regeneration after radiation injury due to a self-renewing capacity [9,10]. It consists of two functional compartments, namely the villi and crypts, which are required for nutrient absorption and self-renewal, respectively. The crypts contain two stem cell populations: an active intestinal stem cell (ISC) population and a +4 position quiescent, reserve ISC population [11]. The active ISC population marked by Lgr5, OLFM4, ASCL2, and SOX9^low^ includes actively dividing stem cells, which are sensitive to ionizing radiation. During radiation injury, the reserve ISC population marked by BMI1, mTERT, KRT19, and SOX9^hi^ survives owing to the radioresistance and regeneration of the epithelium [9]. Recent lineage-tracing approaches have highlighted stem cell heterogeneity and plasticity during the crypt regeneration [11]. However, the effects of particle beam radiation, such as PBT, on ISC populations are not fully elucidated.

During the application of RT to the abdomen or pelvis, normal tissue toxicity in the gastrointestinal (GI) tract is considered a major dose-limiting factor. In view of normal tissue-saving efforts, PBT is a good alternative option. In a clinically relevant normal tissue damage model, jejunal crypt survival after photon and proton irradiation was directly compared at multiple PBT facilities worldwide [12,13,14,15]. Data showed that in vivo RBE ranges between 1.08 and 1.18 at the mid-spread-out-Bragg-peak (SOBP) region, thereby justifying the clinical RBE of 1.1 [4]. However, the cause of the 10% difference in an intestinal crypt survival between the two therapeutic radiation techniques has not yet been determined. In the present study, we performed an in-depth analysis of crypt regeneration in the mouse intestine after high-dose X-ray or proton irradiations to understand the fundamental differences between X-ray- and proton-induced intestinal injury.

## 2. Results

### 2.1. High Doses of Therapeutic Radiation Reduce the Number of Crypts in the Mouse Intestine

**Figure 1:**
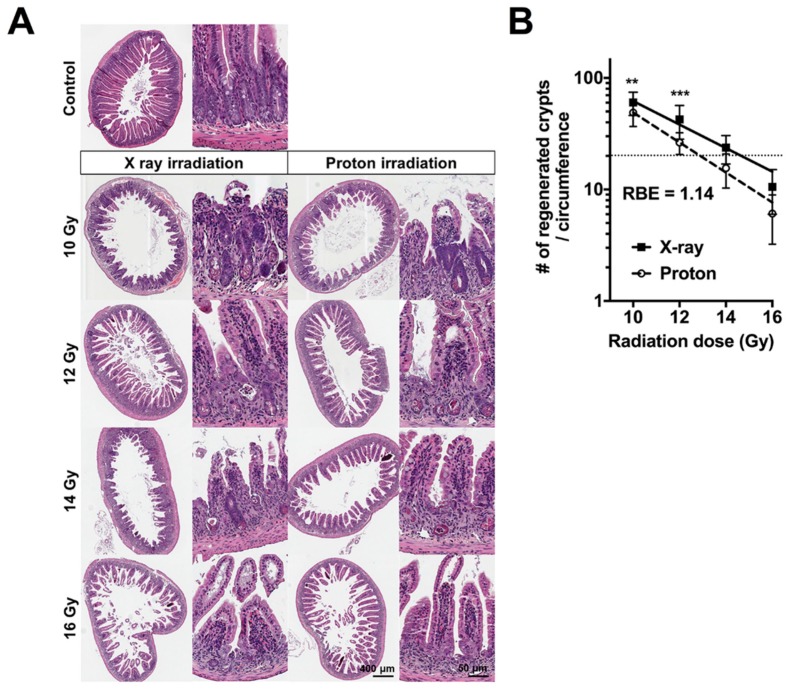
Proton irradiation is more effective in suppressing crypt survival in the mouse jejunum than X-ray irradiation: (**A**) The crypt microcolony assay showed a dose-dependent reduction of the regenerated crypts in X-ray- or proton-irradiated mouse jejunum sections. The abdomens of the anesthetized mice were irradiated with the indicated doses of X-rays or protons. Herein, the representative images of Hematoxylin and Eosin (H&E)-stained transverse sections of jejunum collected at 3.5 days postirradiation are shown. Unirradiated jejunum sections were presented as a control. (**B**) The radiation-dose response curves of mouse jejunum crypts: The data were presented as the mean ± standard deviation of the two independent experiments (*n* = 13). The differences were evaluated by a two-way ANOVA, followed by a Bonferroni posttest; ** *p* < 0.01; *** *p* < 0.001. The relative biological effectiveness (RBE) was defined as the ratio of X-ray dose to proton dose corresponding to 20 regenerated crypts per circumference (dashed line).

Due to the high sensitivity of the mouse intestine to ionizing radiation and its self-renewal capacity, it is used as a standard model system for the assessment of in vivo radiation damage. As a part of the biological QA process at our PBT facility, we compared the biological effectiveness of the two therapeutic radiation beams (6-MV X-rays and 230-MeV protons) in terms of intestinal crypt regeneration. For the crypt microcolony assay, the mice were euthanized, and their abdomens were irradiated with X-rays or proton doses ranging from 10 to 16 Gy. Irradiated jejunum tissues were isolated after 3.5 days and subjected to pathological analysis. Hematoxylin and Eosin (H&E)-stained histological sections showed a normal crypt–villus architecture in the unirradiated control jejunum. However, a severely defective structure was observed in the irradiated jejunum (Figure 1A); a shortening of the villi and a decrease in crypt numbers in both irradiation types were observed dose-dependently. The quantification of crypt numbers showed that protons induced a higher decrease in crypts in the mouse jejunum than X-rays even when the same physical dose was used (Figure 1B). Unirradiated jejunum tissues had crypts of 114.5 ± 8.2 per circumference. The number of crypts significantly decreased with the use of protons compared with X-rays (26.4 ± 5.8 versus 42.5 ± 14.2 at 12 Gy; *p* < 0.001; two-way analysis of variance (ANOVA) with Bonferroni posttests). At our PBT facility, the in vivo RBE was approximately 1.14 based on its standard definition, which is a dose ratio between X-rays and protons corresponding to 20 regenerated crypts per circumference (Figure 1B) and which is very similar to the RBE values in other proton facilities [4,5].

**Figure 2:**
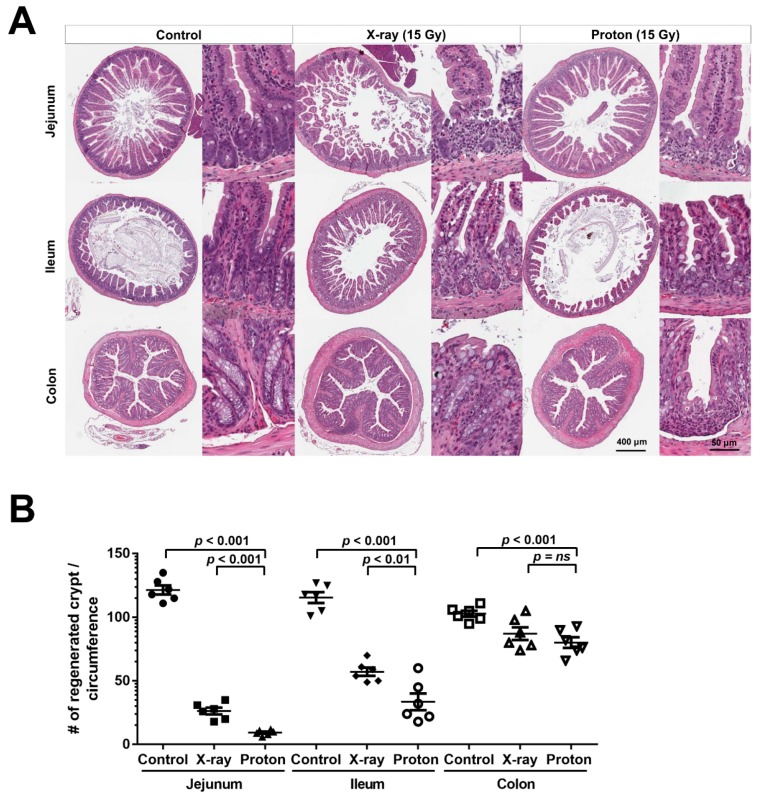
Proton irradiation is more effective in suppressing crypt survival in the mouse small intestine than X-ray irradiation. (**A**) A remarkable decrease in the number of crypts in the small intestine and the colon irradiated with 15 Gy of X-rays or protons compared with unirradiated control tissues: The tissue samples were collected at 3.5 days after irradiation. The representative images of the H&E-stained sections of the jejunum, ileum, and colon are presented herein. (**B**) A quantitation of the crypt numbers per circumference showed that proton irradiation decreased the number of crypts more effectively than X-ray irradiation in both the jejunum and the ileum but not in the colon.

Next, we compared the relative effects of the two therapeutic radiation types on crypt regeneration in the different parts of the mouse intestine. H&E-stained sections prepared under the same conditions showed that 15-Gy X-rays or protons significantly decreased the number of crypts in the jejunum, ileum, and colon, with different sensitivities (Figure 2A). Quantitative analyses showed that the jejunum was the most sensitive organ to radiation injury, followed by the ileum and colon (Figure 2B). This result is in accordance with a previous report showing that colonic epithelial stem cells are more radioresistant than small intestinal cells [16]. Proton irradiation was significantly more effective than X-rays irradiation in the jejunum and ileum but not in the colon (Figure 2B); 15-Gy proton irradiation reduced the fractional crypt survival to a greater extent than X-rays irradiation in the jejunum (92% versus 78%; *p* < 0.001) and the ileum (71% versus 51%; *p* < 0.01). No significant difference between proton and X-ray irradiations was observed in the fractional crypt survival in the colon (22% versus 15%). These data indicated that proton irradiation has a greater effect on crypt survival in the mouse small intestine than X-ray irradiation.

### 2.2. Proton Irradiation Leads to a Higher Decrease in the Numbers of Surviving Crypts in the Jejunum versus X-Ray Irradiation

**Figure 3:**
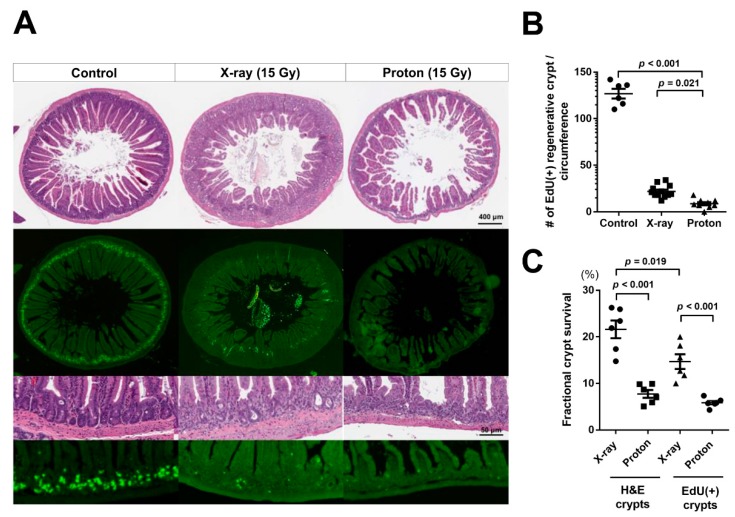
Proton irradiation decreases the number of regenerative crypts in the mouse jejunum to a greater extent than X-ray irradiation: (**A**) The representative H&E-stained (upper panels) and 5-ethynyl-2′-deoxyuridine (EdU)-stained (lower panels) images of the jejunum sections 3.5 days postirradiation with 15 Gy of X-rays or protons are presented herein. Dividing S-phase cells were labelled by EdU administration 2 h prior to euthanasia. (**B**) The quantification showed a significant decrease in the number of EdU-positive crypts by proton irradiation compared with X-ray irradiation. EdU-positive crypt is defined as a crypt that contains five or more EdU-positive cells. (**C**) Fifteen Gy of X-rays or protons induced a more significant decrease in the number of EdU-positive crypts than H&E-stained crypts. The fractional crypt survival was defined as the ratio of crypt numbers in the irradiated sections to those in the unirradiated sections.

Although counting crypts in H&E-stained intestinal tissue sections is a standard in vivo biological dosimetry, whether the crypts have a capacity to regenerate is challenging to identify. To overcome this, we applied 5-ethynyl-2′-deoxyuridine (EdU) staining that can selectively label S-phase dividing cells. EdU was administrated into mice 2 h prior to euthanasia after 3.5 days of 15-Gy X-ray or proton irradiation. In the H&E-stained jejunum sections, the crypts were clearly observed in the unirradiated control group, whereas the number of survived crypts significantly decreased in both the X-ray- and proton-irradiated groups (Figure 3A, upper panels). It was challenging to accurately count the crypts because the villus–crypt architecture was completely disrupted after high-dose radiation (Figure 3A, upper panels). Instead, in the EdU-labelled jejunum sections, EdU-positive crypts were clearly observed around the circumference of the unirradiated control, whereas a few EdU-positive crypts were also found in the irradiated tissues, even under a low magnification (Figure 3A, lower panels). These data indicated that EdU staining may be useful in the visualization of dividing crypts after high-dose irradiation. The quantification data on crypts that contained more than five EdU-positive cells showed that proton irradiation significantly reduced a higher number of Edu-positive surviving crypts than X-ray irradiation (*p* = 0.021) (Figure 3B). The calculation of fractional crypt survival, which is defined as the ratio of crypts numbers in irradiated sections to those in unirradiated sections, confirmed that the survival fraction of EdU-positive crypts was less than that of H&E-stained crypts (Figure 3C). Thus, these data indicated that proton irradiation may injure a higher number of crypt cells that can regenerate than X-ray irradiation.

### 2.3. Proton Irradiation Leads to a Higher Decrease in Quiescent Stem Cells and Progenitor Cells in the Jejunum versus X-Ray Irradiation

**Figure 4:**
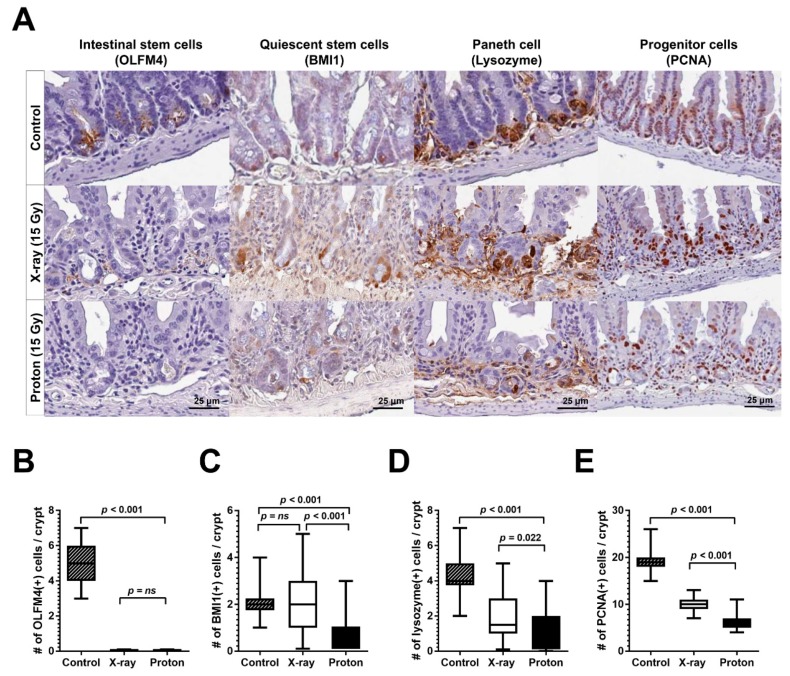
Proton irradiation decreases the population of quiescent stem cells and Paneth cells within the mouse jejunal crypts to a greater extent than X-ray irradiation. (**A**) The representative immunohistochemistry (IHC) results for OLFM4, BMI1, lysozyme, and proliferative cell nuclear antigen (PCNA) in the intestinal crypts 3.5 days postirradiation with 15 Gy of X-ray or proton irradiation are presented herein. The box-and-whisker plots representing distributions of OLFM4-positive (**B**), BMI1-positive (**C**), lysozyme-positive (**D**), and PCNA-positive cells (**E**) within the crypts 3.5 days postirradiation with X-ray or protons.

Crypt regeneration after radiation injury requires stem cells and other cells constituting intestinal crypts. To understand the fundamental difference in crypt regeneration between an X-ray-irradiated and proton-irradiated jejunum, we performed IHC for different crypt cell markers. Jejunum tissue sections were collected 3.5 days after exposure to 15 Gy of X-ray or proton irradiation and were then stained. The mouse jejunum has two stem cell populations—the rapidly dividing ISC and the quiescent, reserve ISC—that differ in terms of radiation sensitivity. IHC showed that the expression of OLFM4, a robust marker for radiosensitive active ISC, was high at the base of the crypts in the unirradiated mouse jejunum and was almost completely eradicated by 15 Gy of both X-ray and proton irradiation (Figure 4A,B). In contrast, the expression of BMI1, a marker for radioresistant, reserve ISC, was not affected by X-ray irradiation but was significantly decreased by proton irradiation (*p* < 0.001; Figure 4A,C). A higher number of Paneth cells, identified with lysozyme IHC, were also eradicated by proton irradiation than X-ray irradiation (*p* = 0.022; Figure 4A,D). The assessment of proliferating cells in the jejunum crypts via proliferative cell nuclear antigen (PCNA) staining showed a significant decrease in PCNA-positive cells in irradiated jejunum crypts; notably, protons irradiation decreased the number of proliferating cells to a greater extent than X-ray irradiation (*p* < 0.001; Figure 4E). These data indicated that proton-induced damage on the BMI1+ quiescent stem cell population may lead to a decrease in the progenitor cell proliferation, resulting in less crypt survival at 3.5 days postirradiation.

### 2.4. Proton Irradiation Leads to a Higher Rate of Apoptotic Cell Death in the Jejunum Crypts versus X-Ray Irradiation

**Figure 5:**
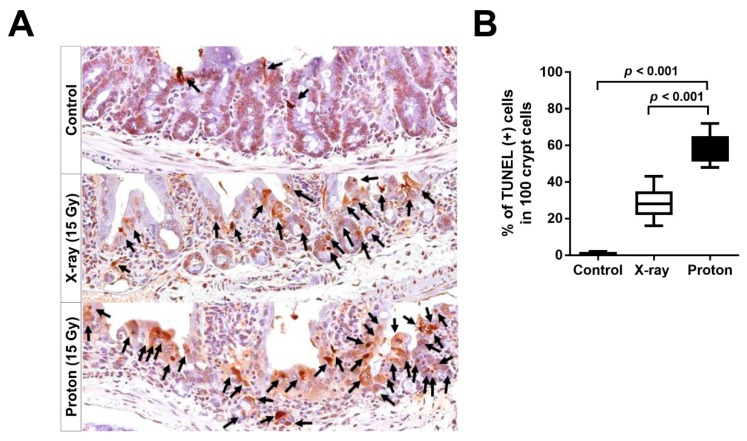
Proton irradiation is more effective in inducing apoptotic cell death in the jejunal crypts than X-ray irradiation. (**A**) The representative terminal deoxynucleotidyl transferase-mediated dUTP nick-end labelling (TUNEL) IHC images of the jejunum sections 3.5 days postirradiation are presented herein. The black arrows indicate TUNEL-positive cells in the crypts. (**B**) The box and whisker plots represent the percentage of TUNEL-positive cells in the irradiated crypts.

A high-dose irradiation leads to the apoptotic death of intestinal epithelial cells, which is mediated by p53 and its downstream effectors, such as the p53 upregulated modulator of apoptosis (PUMA) and p21 [17]. Based on our data indicating that proton irradiation was more effective at decreasing regenerating crypt cells compared than X-ray irradiation, we determined the effects of protons on the apoptosis of crypt cells using the terminal deoxynucleotidyl transferase-mediated dUTP nick-end labelling (TUNEL) staining. In the cross sections of unirradiated control jejunum, a normal crypt architecture with an intact surface epithelium was observed along with just a few TUNEL-positive cells (Figure 5A). In contrast, the tissue sections of X-ray- or proton-irradiated jejunum had a significantly lower number of crypts with an aberrant morphology and a lot of TUNEL-positive cells (Figure 5A). To compare apoptosis induction between X-ray and proton irradiation, we quantified TUNEL-positive cells per 100 crypt cells in the tissue sections. The percentage of TUNEL-positive cells was increased from 0.8 ± 0.6 in unirradiated tissues to 28.7 ± 8.2 by 15 Gy of X-ray irradiation and to 58.5 ± 8.0 by 15 Gy of proton irradiation, and the difference between the two radiation groups was statistically significant (*p* < 0.001; Figure 5B), indicating that protons irradiation may be more effective in increasing apoptotic cell death in the jejunum than X-ray irradiation.

## 3. Discussion

PBT is increasingly used in clinical settings worldwide. However, there are also increasing concerns about its biological uncertainty [6,7,8,18,19,20,21]. Heavy ions, such as carbon ions, consider RBE variations in treatment planning. However, PBT simply uses the generic value of RBE (1.1) at most PBT facilities, including our facility. As summarized by Paganetti et al., the average proton RBE at the mid-SOBP obtained from numerous experiments was approximately 1.2 in vitro and 1.1 in vivo, which provides the rationale for the current clinical use of the generic RBE value [5]. However, accumulating evidence indicates that the existing uncertainty in proton RBE should not be ignored in some clinical cases [22]. LET sharply increases at the distal end of the SOBP, leading to an increase in RBE, which may induce a significant toxicity in organs or normal tissues in proximity to tumors [23]. The dependency of RBE on the tissue-specific α/β ratio and genetic factors is well-recognized [24] but not considered as an RBE-weighting factor in clinical settings. Thus, a more detailed mechanistic study using in vivo model systems is required to solve the issues related to uncertainty in proton RBE.

Regarding tumor response, an in vivo proton RBE estimation was acquired from xenografted tumor models. When tumor growth delay was used as a biological endpoint, the in vivo proton RBE ranged from 1.1 to 1.4, depending on the tumor type and experimental settings [25,26,27]. Regarding normal tissue response, there are numerous experimental results about early and late tissue responses in in vivo animal models. The skin is one of the good model systems for this purpose; an RBE measurement with acute skin reaction, skin epilation, and late skin contraction in mice revealed RBE values ranging from 1.00 to 1.25 for a skin reaction [28,29,30,31]. In terms of quantitative metrics, intestinal crypt cell survival assay is an excellent method to compare the relative normal tissue effect of different radiation modalities. Guelette et al. have shown that the RBE for a high single-dose irradiation at the mid-SOBP of a 200 MeV proton beam was 1.15 based on 20 surviving crypts [14]. In the following studies, these authors have shown that proton RBE for crypt regeneration was independent of fractionation schedules [32] and the RBE of a scanning proton beam at the Paul Scherrer Institute in Villigen, Switzerland, slightly increased from 1.11 to 1.21 toward the end of the SOBP [15]. Our in vivo RBE of 1.14 was extremely similar to those from other PTB centers, and in this study, we first determined the in vivo RBE at our PTB facility as part of the biological QA process.

For an in vivo RBE calculation, intestinal tissue responses to the two types of radiation are quantitated by manually counting the number of crypts in H&E-stained tissue sections [4]. However, there is a disadvantage; counting H&E stained crypts may not be accurate for RBE because their morphology significantly changes after an exposure to high-dose radiation. In this study, we used an EdU staining assay, which can facilitate the labelling of actively dividing cells in the jejunum crypts by incorporating EdU into the DNA of S-phase cells. A pulsed assay using S-phase biomarkers such as EdU or BrdU (5-bromo-2′-deoxyuridine) is intensively used in intestinal regeneration studies after radiation treatment. The assay was applied to show the radioprotective function of the CDK4/6 inhibitor or histamine [33,34]. In addition, it was useful in dissecting apoptosis-related signaling pathway, such as p53 and PUMA [17,35]. Particularly, the BrdU-labeling kinetics was important for elucidating mechanisms underlying differential radiosensitivity among stem cell populations that are related to DNA damage pathways and cell cycle regulation [16,17,36]. In this study, EdU staining revealed that proton irradiation decreased a higher number of regenerating jejunum crypts than X-ray irradiation. Considering that Edu-positive crypts could repopulate and regenerate an entire villi–crypt architecture, our data suggest that proton irradiation may be more effective in eradicating regenerating jejunum crypts than X-ray irradiation.

Considering that the difference in regenerating jejunum crypts may be correlated to stem cell populations, we determined the radiation effect on the distribution of functionally distinct cell populations in the mouse jejunum crypts. The responses of intestinal epithelial cell populations to radiation injury and their representative markers are well-characterized in elegant genetic lineage tracing experiments [9,10,37]. Mouse intestinal epithelium can regenerate after total body irradiation with doses below 14 Gy. However, it can be affected by irradiation with doses above 15 Gy [38,39]. Our IHC data showed that radiosensitive, active ISC marked by OLFM4 were almost completely depleted after 15 Gy of X-ray or proton irradiation. However, the radioresistant, reserve ISC population, as marked by BMI1, was significantly diminished by 15-Gy proton irradiation but not by X-ray irradiation. Although most active ISC underwent apoptosis within 24 h after exposure to 12 Gy of X-rays, a subset of ISC generally survived due to an efficient DNA damage repair capacity and then repopulated after 48 h [39]. The slow-cycling ISC subpopulation, which was characterized by a high expression of Cdkn1a or Mex3a, was also resistant to 12 Gy irradiation and regenerated crypts [40,41]. Our dose of 15 Gy was strong enough to deplete the most active ISC population (Figure 4B). However, we cannot exclude the possibility that these radioresistant subsets of active ISC still survived after 15-Gy proton irradiation. A subpopulation of BMI1+ stem cells marked by KRT19 or KLF4 was found to be resistant to ionizing radiation, and it contributes to the entire crypt regeneration after a radiation injury [42,43]. In addition to BMI1+ cells, proton irradiation induced a higher decrease in Paneth cells than X-ray irradiation (Figure 4D). Based on these data, there are two possible mechanisms underlying the relative effectiveness of proton irradiation over X-ray irradiation in terms of crypt survival. One mechanism is that proton irradiation exerted a stronger ablation effect directly on the BMI1+ reserve ISC than X-ray irradiation, which prevents the radioresistant population from regenerating the crypts. The other mechanism is that proton irradiation destroyed the stem cell niche, including Paneth cells, resulting in a delay in Lgr5+ ISC repopulation and differentiation. Either of these two mechanisms or the combination of both may lead to a more significant decrease in PCNA expression (Figure 4E) and EdU-positive crypts (Figure 3A) by 15-Gy proton irradiation. The diminished proliferation may be reversely correlated with an enhanced apoptosis based on TUNEL staining (Figure 5).

Regenerative processes after RT comprise three phases; the apoptotic phase (the first 2 days), the proliferative phase (2–4 days), and the normalization phase (4–7 days) [9]. In our study, only a few survived cells (EdU-positive) entered the proliferative phase and a lot of apoptotic cells (TUNEL-positive) were detected in the irradiated crypts. Radiation-induced apoptosis is dependent on p53 and PUMA through the mitochondrial pathway during an intestinal radiation injury [17]. Cytochrome c release from mitochondria leads to the multimerization of Bax, resulting in increasing caspase 3 activity. The pharmacological inhibition of p53-dependent apoptosis protects intestinal stem cells from radiation injury [44]. Previous studies have shown that proton irradiation compared with photon irradiation has a stronger effect on apoptotic cell death in glioma stem cells and other cancer cells, including hepatocellular carcinoma cells, via the caspase-dependent pathway [25,45,46,47]. Mechanistically, proton irradiation significantly increases the production of reactive oxygen species and the expression of proapoptotic genes, such as Bax and p21, compared with photon irradiation [25,46,47]. Thus, we speculated the enhanced apoptosis in jejunum crypts induced by proton irradiation may be attributed to the p53-dependent apoptosis pathway. There is need for more detailed studies on which apoptotic components are induced by proton versus photon irradiation in the intestinal radiation injury.

Normal tissue toxicity is a major dose-limiting factor during RT. RT on tumors in the abdominal cavity or pelvis increases the risk of bowel damage, leading to GI symptoms. Recent advances in precise beam delivery techniques, such as intensity modulated radiation therapy and particle therapy, improved normal tissue sparing. The superior dose distribution of PBT indicates that patients who receive PBT in prostate or pelvic nodes may experience less acute GI toxicity than those who receive conventional RT. However, our data indicated that proton irradiation may be more effective in inducing severe bowel toxicity by depleting the quiescent stem cell population. This study used large doses (10–18 Gy) of proton beams in a single fraction at the mid-SOBP. However, other clinical situations, including conventional fractionation (1.8–2 Gy) and the distal edge of the SOBP may also potentially increase the overall RBE, particularly in normal tissues. Thus, radiation oncologists must pay more attention regarding a reduction in the proton doses to the bowel during PBT. From another perspective, our findings may be applicable to the eradication of intestinal cancer stem cells using PBT. KRT19+/Lgr5− cells may be cancer-initiating and radioresistant, making them functionally distinct from the radiosensitive Lgr5+ ISC population [42]. If protons are proven to be effective in eradicating those radioresistant cancer stem cell populations, PBT may reduce the risk of colorectal cancer recurrence compared with conventional X-ray therapy.

## 4. Materials and Methods

### 4.1. Mice and Irradiation Procedures

The procedures for all animal experiments were conducted in accordance with all appropriate regulatory standards under protocol (ID: 20160125002; approval date: 18 February 2016) reviewed and approved by the Institutional Animal Care and Use Committee (IACUC) of Samsung Biomedical Research Institute (SBRI) at Samsung Medical Center in Seoul, South Korea. C57BL/6J mice were purchased from Orient Bio (Seongnam, South Korea). All animal experiments were performed in accordance with relevant guidelines and regulations. The mice were maintained in a 12-h light/12-h dark cycle under specific pathogen-free conditions and were fed ad libitum in the Laboratory Animal Research Center of SBRI. Ten to fourteen-week-old mice were used in this study and were randomly divided into three groups as follows: controls, X-ray irradiation, and proton irradiation.

X-ray and proton irradiations were performed with a Varian Clinac 6EX linear accelerator (Varian, Medical Systems, Palo Alto, CA, USA) and a Sumitomo proton therapy system (Sumitomo Heavy Industries, Tokyo, Japan), respectively, at Samsung Medical Center. The mice were anesthetized by an intraperitoneal injection of 30 mg/kg zolazepam/tiletamine and 10 mg/kg xylazine prior to irradiation. For X-ray irradiation, the mice were placed under a 2-cm-thick water-equivalent bolus with a source-to-surface distance of 100 cm and a field size of 32 cm × 7 cm. Proton beam irradiation was performed with the wobbling method, a mode of the passive scattering techniques [48]. The maximum energy of proton beam was 230 MeV, and its range was 22.8 cm. The SOBP with a 10-cm width was generated using ridge filters. The mice were placed into a special jig and positioned at the middle of the SOBP (17 cm), which was achieved by placing a water-equivalent solid phantom on the jig. The abdomen area, including the small/large bowl and colon was irradiated, with graded single doses of 6-MV X-rays and 230-MeV protons at a dose rate of 3.96 and 2.14 Gy per minute, respectively. The X-ray absolute dose was calibrated according to the TG-51 protocol and verified with the Gafchromic film with a 1% accuracy. The proton absolute dose was determined and verified according to the TRS-398 protocol with a 1% accuracy. The range and SOBP width of the proton beams were measured using a Zebra multilayer ionization chamber (IBA dosimetry, Schwarzenbruck, Germany).

### 4.2. Crypt Microcolony Assay

To compare the dose-dependent radiation damage, the mice were exposed to 10, 12, 14, and 16 Gy of X-ray or proton irradiation and sacrificed after 84 h (3.5 days) [49]. The number of surviving crypts per circumference of the jejunum was identified using H&E-stained sections. The unirradiated jejunum sections were presented as controls. To assess the location-dependent radiation damage, the mice were exposed to 15 Gy of X-ray or proton irradiation and sacrificed after 84 h (3.5 days) [49]. The number of surviving crypts per circumference of the jejunum, ileum, and colon, respectively, were identified using H&E-stained sections.

### 4.3. Edu Assay

Two hours before sacrifice, the mice were intraperitoneally injected with 200 μg of EdU (Sigma-Aldrich, St. Louis, MO, USA) dissolved in phosphate-buffered saline (PBS). All mice were sacrificed, and their intestines were harvested 84 h after injection. The incorporation of EdU into the DNA was detected using the Click-iT™ EdU Alexa Fluor^®^ 488 Imaging kit (Thermo Fisher Scientific, Waltham, MA, USA). The EdU-positive surviving crypt was defined as a crypt containing five or more EdU-positive cells [50]. The number of surviving crypts per circumference of the jejunum was identified.

### 4.4. Immunohistochemistry

Immunohistochemistry (IHC) was performed as previously described [25]. Briefly, the 4-μm thick sections were deparaffinized in xylene, rehydrated in graded alcohol, and washed with 0.01-M PBS (pH 7.4). After the epitope retrieval with a citrate buffer (pH 6.0; Dako, Carpinteria, CA, USA) and the blocking with a blocking solution (Dako), the tissue sections were incubated with anti-OLFM4 (1:400 dilution, Cell Signalling Technology, Danvers, MA, USA), anti-PCNA (1:100 dilution, Abcam, Cambridge, UK), anti-lysozyme (1:3000 dilution, Abcam), and anti-BMI1 (1:400 dilution, Abcam) antibodies overnight at 4 °C. After washing with PBS, the sections were incubated for 30 min with horseradish peroxidase-conjugated secondary antibodies (Dako) and the antigen–antibody interaction was visualized using the chromogenic substrate 3,3′ diaminobezidine (DAB; Dako).

### 4.5. TUNEL Assay

Apoptosis-associated DNA fragmentation was detected via TUNEL using the In Situ Cell Death Detection Kit (Sigma-Aldrich). TUNEL was carried out according to the manufacturer’s instructions. Positive control sections were incubated with 10 U/mL of recombinant deoxyribonuclease I solution, and the negative control was processed in the same manner, albeit with the omission of the terminal transferase enzyme. The percentage of TUNEL-positive cells were quantified by counting 100 cells from randomly selected fields.

### 4.6. Statistics

The differences between the experimental groups was analyzed using the Mann–Whitney U test or a two-way ANOVA with the Bonferroni posttest. GraphPad Prism v7.04 (GraphPad Software, La Jolla, CA, USA) was used in the analyses. A *p* value < 0.05 was considered statistically significant.

## 5. Conclusions

The biological effect of proton irradiation versus X-ray irradiation on crypt regeneration in the mouse intestine was analyzed in detail. Our findings showed that proton irradiation had a more direct effects on the ISC population than X-ray irradiation, which may increase the risk of GI toxicity during PBT.

## Abbreviations

EdU	5-ethynyl-2′-deoxyuridine
GI	gastrointestinal
H&E	haematoxylin and eosin
ISC	intestinal stem cell
IHC	immunohistochemistry
OLFM4	Olfactomedin 4
PBT	proton beam therapy
RBE	relative biological effectiveness
RT	radiation therapy
SOBP	spread-out-Bragg-peak
TUNEL	terminal deoxynucleotidyl transferase-mediated dUTP nick-end labelling
